# Anomalous Water-Sorption Kinetics in ASDs

**DOI:** 10.3390/pharmaceutics14091897

**Published:** 2022-09-07

**Authors:** Dominik Borrmann, Andreas Danzer, Gabriele Sadowski

**Affiliations:** Laboratory of Thermodynamics, Department of Chemical and Biochemical Engineering, TU Dortmund University, Emil-Figge-Str. 70, 44227 Dortmund, Germany

**Keywords:** relaxation, diffusion, swelling controlled, water-sorption kinetics, ASDs

## Abstract

Anomalous water-sorption kinetics in amorphous solid dispersions (ASDs) are caused by the slow swelling of the polymer. In this work, we used a diffusion–relaxation model with the Williams–Landel–Ferry (WLF) equation and the Arrhenius equation to predict the anomalous water-sorption kinetics in ASDs of poly(vinyl-pyrrolidone)-*co*-vinyl-acetate (PVPVA) and indomethacin (IND) at 25 °C. These predictions were based on the viscosities of pure PVPVA and pure IND, as well as on the water-sorption kinetics in pure PVPVA. The diffusion–relaxation model was able to predict the different types of anomalous behavior leading to a qualitative and quantitative agreement with the experimental data. Predictions and experiments indicated more pronounced anomalous two-stage water-sorption behavior in the ASDs than in pure PVPVA. This was caused by a higher viscosity of glassy ASD–water mixtures compared to glassy PVPVA–water mixtures at the same distance from their glass transition temperature. These results suggest that this ASD swells more slowly than the polymer it is composed of. The modeling approach applied in this work can be used in the future for predicting diffusion-controlled release behavior or swelling-controlled release behavior of ASDs.

## 1. Introduction

The poor water solubility of orally administered active pharmaceutical ingredients (APIs) leads to a low bioavailability. Therefore, a polymer is often combined with an API to form an ASD, where the polymer serves as a stabilization and dispersion agent. As a result, the API release from this ASD is significantly improved compared to the pure crystalline API. However, due to its polymer content, the release behavior of an ASD is overlaid by water sorption into the ASD during dissolution in aqueous media and the subsequent swelling of the ASD. Peppas et al. [1,2,3,4,5] found two primary controlling release mechanisms: diffusion-controlled release and swelling-controlled release [1].

Diffusion-controlled release can be modeled using Fick’s laws of diffusion. However, swelling impacts the release from the polymer-based formulations leading to a non-Fickian diffusion behavior [3,6,7,8]. Swelling-controlled release [1] is suitable to control API release, as shown by Peppas et al. [9,10] for hydroxy-propyl-methylcellulose discs loaded with buflomedil pyridoxal phosphate.

The physical phenomena leading to diffusion-controlled or swelling-controlled release are not well understood. Ewing et al. [6] investigated the dissolution of ASDs containing the API aprepitant and either poly(vinyl-pyrrolidone) PVP or soluplus and mapped their dissolution profiles via spectroscopic imaging. The hydrophilicity of the polymers and their ASDs significantly impacted their dissolution rate, API release, and swelling tendency. The dissolution rate of pure soluplus was significantly lower than that of pure PVP, and soluplus formed a slowly swelling layer which PVP did not. In contrast, ASDs from either PVP or soluplus formed swelling layers, and the API release from the PVP-based ASD was drastically reduced compared to that of the soluplus-based ASDs.

Existing theories for predicting the dissolution of polymers and API release from polymer-based systems were summarized by Miller-Chou and Koenig [7]. These theories include stress-relaxation theories to account for the influence of polymer swelling on diffusion. The slow swelling of the polymer is explained by its resistance to move generating a mechanical stress. The decrease in this mechanical stress depends on time and influences the diffusion of both polymer and solvent, explaining a diffusion behavior in polymer-based systems that deviates from Fick’s laws [11], which is often called anomalous sorption behavior. As API dissolution is influenced by water sorption into the ASD, modeling the anomalous water-sorption kinetics in ASDs generates insight into its dissolution kinetics in aqueous media.

Fiornasiero et al. [12] reported anomalous water-sorption kinetics in PVP films at 25 °C. They found significant time lags leading to a sigmoidal course in water sorption, which is uncommon for diffusion-controlled sorption. The time lag increased with relative humidity (RH) and was highest for an RH step from 0 to 0.72. Our previous work [13] also reported sigmoidal water-sorption kinetics in PVP and PVPVA at 25 °C but using successive, step-wise RH changes. The sigmoidal water-sorption kinetics was most prominent in the RH step from 0.6 to 0.75, in which PVP–water mixtures transitioned from being glassy to being rubbery.

In a subsequent study [14], we observed even stronger anomalous water-sorption kinetics in PVP-IND and PVPVA-IND ASDs. As a result, the water-uptake rate of these ASDs decreased with increasing drug load and was lower than in pure PVP and PVPVA. Moreover, the sharp upward curvature of the sigmoidal water-sorption kinetics of the ASDs near their glass transition was more prominent than the sigmoidal water-sorption kinetics of pure PVP and PVPVA. Since sorption behavior becomes more anomalous the slower the system swells, it is reasonable to conclude that the ASD tendency to slowly swell increases with increasing drug load. Zordan et al. [15] studied PVP-based IND ASDs, which also showed decreased IND release and slower swelling for higher drug loads.

Modeling the water-sorption kinetics in an ASD while considering the slow swelling of the ASD has not been considered yet. This work uses a diffusion model based on a stress-relaxation theory to predict the anomalous water-sorption kinetics observed in PVPVA and PVPVA-IND ASDs using the viscoelastic properties of the polymer and the ASD, respectively. For that purpose, we applied the temperature–humidity–drug load correspondence principle by Wolbert et al. [16] (analogous to the well-known time–temperature principle [17]) to predict the viscosities of rubbery ASD–water mixtures solely on the basis of the temperature dependency of the pure-polymer viscosity. Moreover, this work extends this principle by predicting the viscosity of glassy ASD–water mixtures.

## 2. Modeling

### 2.1. Diffusion–Relaxation Model

In a previous study, we derived a diffusion model from the stress-relaxation theory [11]. This generalized model is referred to as the diffusion–relaxation model in this work. It describes the sorption of any solvent in any polymer system, and it was used in this work to model anomalous water-sorption kinetics in the polymer and in the ASD. The change in water concentration was described using Equation (1).

(1) $$\frac{\partial \bar{\rho_{w}}}{\partial t}=\frac{\partial }{\partial \bar{z}}\left(\bar{\rho_{w}}\;{\left(1+\epsilon \right)}^{-2}\frac{{Đ}_{w}^{\prime \prime }}{1-w_{w}}\;\left(\frac{v_{0w}M_{w}}{RT}{\left(\frac{\partial \sigma }{\partial \bar{z}}\right)}_{T}+\frac{1}{a_{w}}\frac{\partial a_{w}\;}{\partial \bar{\rho_{w}}}{\left(\frac{\partial \bar{\rho_{w}}}{\partial \bar{z}}\right)}_{T}\right)\right),$$

where $\bar{z}$ is a transformed spatial coordinate along with the thickness $L_{0}$ of the dry film. $\bar{\rho_{w}}$ is the transformed water concentration, which is the water mass $m_{w}$ relative to the volume $V_{0}$ of the dry film, and t is the time. $v_{0w}$ is the specific volume of water, $w_{w}$ is the weight fraction of water in the polymer or ASD, and $M_{w}$ is the molar mass of water. ${Đ}_{w}^{\prime \prime }$ is the segmental Stefan–Maxwell diffusion coefficient of water in the film, and $a_{w}$ is the water activity. R is the ideal gas constant, and T is the absolute temperature. The strain $\epsilon =\bar{\rho_{w}}v_{0w}$ was expressed in terms of the water concentration assuming volume additivity. The decrease in stress σ was modeled by a Maxwell element leading to Equation (2).

(2) $$\frac{\partial \sigma }{\partial t}=\frac{\partial \epsilon }{\partial t}E-\sigma \frac{E}{\eta },$$

where E is the elastic modulus reached when time approaches zero, and η is the viscosity reached when time approaching infinity (Newtonian plateau). It is worth noting that these quantities do not depend on time nor on the rate of deformation. Thus, E in Equation (2) describes the purely elastic response of the film, while η describes the purely viscous response of the film. Equations (1) and (2) were solved by discretizing the film into finite volume elements, leading to $n_{z}$ volume elements and an additional $n_{z}+1$ element, the latter being referred to as the surface element. The evolution of the water concentration ${\bar{\rho_{w}}}^{n_{z}+1}$ in the surface element was described according to Equation (3),

(3) $$\frac{\partial {\bar{\rho_{w}}}^{n_{z}+1}}{\partial t}=\frac{\sigma^{n_{z}+1}\frac{E}{\eta }\;\frac{v_{0w}}{RT}}{\frac{1}{a_{w}^{n_{z}+1}}\frac{\partial a_{w}\;}{\partial \bar{\rho_{w}}}+\frac{v_{0w}^{2}M_{w}E}{RT}},$$

depending on the stress $\sigma^{n_{z}+1}$ in the surface element, which is given in Equation (4).

(4) $$\sigma^{n_{z}+1}=\frac{RT}{v_{0w}M_{w}}\ln\left(\frac{a_{w}^{\infty }}{a_{w}^{n_{z}+1}}\right),$$

where $a_{w}^{n_{z}+1}$ is the water activity in the surface element, and $a_{w}^{\infty }$ is the water activity at the end of the sorption curve (in equilibrium).

The initial water concentration ${\bar{\rho }}_{w}^{+}={\bar{\rho_{w}}}^{n_{z}+1}\left(t=0\right)$ in the surface element was obtained through Equation (5).

(5) $${\bar{\rho }}_{w}^{+}={\bar{\rho }}_{w}^{0}+\frac{RT}{v_{0w}^{2}M_{w}E}\ln\left(\frac{a_{w}^{\infty }}{a_{w}^{+}}\right),$$

where ${\bar{\rho }}_{w}^{0}$ is the water concentration corresponding to the start of the water-sorption curve. The water activity $a_{w}^{+}$ corresponds to the water concentration ${\bar{\rho }}_{w}^{+}$. The water activity $a_{w}$ as a function of the transformed water concentration $\bar{\rho_{w}}$ was linearly approximated between the sorption curve’s start and endpoint using Equation (6).

(6) $$a_{w}\left(\bar{\rho_{w}}\right)=\frac{a_{w}^{\infty }-a_{w}^{0}}{{\bar{\rho }}_{w}^{\infty }-{\bar{\rho }}_{w}^{0}}\left(\bar{\rho_{w}}-{\bar{\rho }}_{w}^{0}\right)+a_{w}^{0}=\frac{\partial a_{w}}{\partial \bar{\rho_{w}}}\left(\bar{\rho_{w}}-{\bar{\rho }}_{w}^{0}\right)+a_{w}^{0},$$

where ${\bar{\rho }}_{w}^{0}$ and ${\bar{\rho }}_{w}^{\infty }$ are the transformed water concentrations measured at the start and the end of the water-sorption curves, respectively. $a_{w}^{0}$ is the water activity at the start of the water-sorption curve. The water activities $a_{w}^{0}$ and $a_{w}^{\infty }$ at the start and the end of the sorption curve, respectively, directly correspond to the RHs at the start and the end of each sorption step.

Transformed water concentrations $\bar{\rho_{w}}$ were related to the water weight fractions $w_{w}$ using the specific volume $v_{0}=\frac{V_{0}}{m_{0}}$ of the dry film with $m_{0}$ being the mass of the dry film, as seen in Equation (7).

(7) $$\bar{\rho_{w}}v_{0}=\frac{w_{w}}{1-w_{w}},$$

We used Equation (7) to relate water weight fraction $w_{w}$ and transformed water concentration $\bar{\rho_{w}}$ to one another. In this way, ${\bar{\rho }}_{w}^{0}$ and ${\bar{\rho }}_{w}^{\infty }$ were obtained from the water weight fractions $w_{w}^{0}$ at the start and at the end $w_{w}^{\infty }$ of the water-sorption curve, respectively. The specific volume $v_{0}=w_{0a}v_{0a}+w_{0a}v_{0p}$ of the dry films were calculated using the drug load $w_{0a}$ and the polymer load $w_{0p}$, as well as the specific volumes $v_{0a}$ of the API and $v_{0p}$ of the polymer.

Equations (1) and (2) were solved simultaneously with Equation (3) as a boundary condition. For more information about the diffusion–relaxation model and its numerical solution, the reader is referred to our previous work [11].

### 2.2. Viscosity Modeling

**Figure 1:**
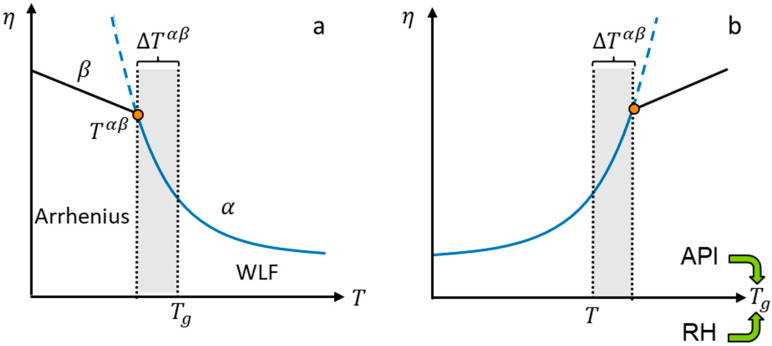
(**a**) Schematic representation of the temperature dependency of viscosity. The solid lines describe the viscosity of a polymer or API modeled via the WLF equation or Arrhenius equation. The dashed line is the extrapolation of the WLF equation to the Arrhenius region. The point marks the temperature $T^{\alpha \beta }$ at which the temperature dependency switches from WLF to Arrhenius behavior. The distance $\Delta T^{\alpha \beta }$ of the switching temperature $T^{\alpha \beta }$ to the glass transition temperature $T_{g}\;$ is indicated as a gray box. (**b**) Schematic representation of viscosity as a function of the glass transition temperature $T_{g}$. Drug load and relative humidity (RH) alter the glass transition temperature of the mixture and decrease its viscosity. The glass transition occurs when the glass transition temperature $T_{g}$ is equal to the temperature T.

The viscosity modeling is visualized in Figure 1. The WLF equation was used to model the temperature dependency of the viscosity of rubbery PVPVA–water and ASD–water mixtures (Figure 1a). However, it is well known that the WLF equation overestimates the viscosities of glassy systems, which was demonstrated for various materials by Hiki et al. [18,19]. This is because the global molecular motions (α-relaxation) become very slow in glassy systems, whereas localized conformational motions (β-relaxation) become much faster [20]. However, these localized motions predominate the relaxation in glassy systems only at temperatures significantly lower than $T_{g}$, which causes a change in the viscosity–temperature behavior at a temperature lower than $T_{g}$. We refer to this temperature as the switching temperature $T^{\alpha \beta }$ and define the distance of the switching temperature $T^{\alpha \beta }$ to the glass transition temperature $T_{g}$ as $\Delta T^{\alpha \beta }=T^{\alpha \beta }-T_{g}$.

Upon isothermal water sorption of an ASD, the viscosity decreases with increasing water concentration [16]. This decrease in viscosity happens due to the plasticization effect of water and the API on the polymer reducing the glass transition temperature $T_{g}$ of the mixture. It can be assumed [16] that reducing the glass transition temperature $T_{g}$ has a similar effect on the viscosity to increasing the temperature T. Thus, the course of the viscosity with the glass transition temperature $T_{g}$ is mirrored in its temperature dependency (Figure 1b). The glass transition occurs when the glass-transition temperature $T_{g}$ is equal to the system temperature T. Since $T^{\alpha \beta }=T_{g}+\Delta T^{\alpha \beta }$, the switching temperature $T^{\alpha \beta }$ of the mixture decreases with decreasing glass transition temperature.

The viscosity η as a function of $T_{g}$ was described according to the WLF equation in Equation (8) (see Wolbert et al. [16]).

(8) $$\log_{10}\frac{\eta }{\eta_{ref}}=\frac{C_{1}\left(\left(T-T_{g}\right)+\left(T_{g0p}-T_{ref}\right)\right)}{C_{2}+\left(\left(T-T_{g}\right)+\left(T_{g0p}-T_{ref}\right)\right)}\;T\geq T^{\alpha \beta },$$

where $\eta_{ref}$ is the viscosity at a reference temperature $T_{ref}$. $T_{g}$ is the glass transition temperature of the mixture at temperature *T*. $C_{1}{\;\mathrm{and}\;C}_{2}$ are the WLF parameters of the polymer, and $T_{g0p}$ is the glass transition temperature of the polymer. Equation (8) is valid for temperatures T higher than $T^{\alpha \beta }$. The Arrhenius equation (Equation (9)) was used to describe the viscosity when T is lower than $T^{\alpha \beta }$ (Equation (9)).

(9) $$\log_{10}\frac{\eta }{\eta \left(T^{\alpha \beta }\right)}=-\frac{E_{A}}{2.303\cdot R}\;\left(\frac{1}{T_{g}}-\frac{1}{T-\Delta T^{\alpha \beta }}\right)\;T<T^{\alpha \beta },$$

where $E_{A}$ is the activation energy for relaxation in the ASD. The viscosity $\eta \left(T^{\alpha \beta }\right)$ is that obtained from the WLF equation (Equation (8)) when $T^{\alpha \beta }=T$ to ensure the continuity of the expressions Equations (8) and (9).

The activation energy $E_{A}$ of the mixture and $\Delta T^{\alpha \beta }$ were predicted via Equations (10) and (11).

(10) $$E_{A}=w_{0p}E_{Ap}+w_{0a}E_{Aa},$$

(11) $$\Delta T^{\alpha \beta }=w_{0p}\Delta T_{0p}^{\alpha \beta }+w_{0a}\Delta T_{0a}^{\alpha \beta },$$

where $w_{0p}$ is the polymer load and $w_{0a}$ is the drug load of the ASD. $E_{Ap}$ is the activation energy for relaxation in the polymer, and $E_{Aa}$ is the activation energy for relaxation in the API. $T_{0p}^{\alpha \beta }$ is the switching temperature of the polymer with its difference $\Delta T_{0p}^{\alpha \beta }=T_{0p}^{\alpha \beta }-T_{g0p}$ to the glass transition temperature $T_{g0p}$ of the polymer. $T_{0a}^{\alpha \beta }$ is the switching temperature of the API with its difference $\Delta T_{0a}^{\alpha \beta }=T_{0p}^{\alpha \beta }-T_{g0a}$ to the glass transition temperature $T_{g0a}$ of the API.

The glass transition temperature $T_{g}$ of the mixture was predicted using Equation (12).

(12) $$T_{g}=\frac{K_{a}w_{a}T_{g0a}+K_{p}w_{p}T_{g0p}+w_{w}T_{g0w}}{K_{a}w_{a}+K_{p}w_{p}+w_{w}},$$

where $K_{a}$ is the Gordon–Taylor constant of the API–water mixture, and $K_{p}$ is the Gordon–Taylor constant of the polymer–water mixture. $T_{g0w}$ is the glass transition temperature of water.

### 2.3. Model Parameters

The specific volumes of water [21] $v_{0w}=1.003\frac{{\mathrm{cm}}^{3}}{g}$, of PVPVA [22] $v_{0p}=0.8474\;\frac{{\mathrm{cm}}^{3}}{g}$, and of IND [23] $\;v_{0a}=0.7576\;\frac{{\mathrm{cm}}^{3}}{g}$ were calculated from their pure component densities taken from the indicated sources. The Gordon–Taylor constant of PVPVA–water mixtures ($K_{p}=0.3$) was taken from previous work [14], while that of IND–water ($K_{a}=0.11)$ was taken from Zografi et al. [24]. The glass transition temperatures of water [25] $\;(T_{g0w}=136\;K)$, of PVPVA [22] $\left(T_{g0p}=383.9\;K\right)$, and of IND [26] $\left(T_{g0a}=317.6\;K\right)$ were also taken from the literature. The parameters used for the diffusion–relaxation model used in this work are displayed in Table 1.

The parameters $E,\eta_{ref}$, $C_{1}$, and $C_{2}$ are measurable through rheological measurements of the polymer. The parameters $C_{1}$ and $C_{2}$ of PVPVA–water mixtures at the reference temperature $T_{ref}=423.15\;K$ were determined by Wolbert et al. [16]. They used a shear-oscillatory rheometer to determine the zero-shear viscosities of PVPVA–water mixtures (shear-rate-independent viscosity at the Newtonian plateau) with a reference zero-shear viscosity of 0.61 × 10^5^ Pa·s at the reference temperature $T_{ref}=423.15\;K$. In this study, we modeled the elongation of a film during water sorption, which is the equivalent of drawing the sample in the tensile direction. As a result, we approximated the viscosity $\eta_{ref}$ by scaling the zero-shear viscosity at the reference temperature by a factor of 3, which is a common heuristic for relating shear and elongational viscosities in Newtonian fluids (see Mezger [30]). This is valid as the viscosity used in Equation (2) is strain-rate independent and, therefore, Newtonian. The elastic modulus of E=4 GPa was fitted to the low-temperature storage modulus of PVP taken from Cassu and Felisberti [29] as shown in Figure S1 of the Supplementary Materials. We assumed that the elastic modulus of E was the same for PVPVA and PVP.

The parameters $E_{Aa}$ $E_{Ap}$, $\Delta T_{0a}^{\alpha \beta }$, and $\Delta T_{0p}^{\alpha \beta }$ can be determined from the viscosities or relaxation times of the polymer and the API. The difference $\Delta T_{0a}^{\alpha \beta }$ for IND was taken from Andronis and Zografi [28], who observed a deviation of IND relaxation times from WLF behavior at about 20 K below its glass transition temperature.

As the focus of this study was on predicting anomalous water-sorption behavior, we used a constant ${Đ}_{w}^{\prime \prime }$ for all predictions although we know that it depends on water concentration and differs for glassy and rubbery systems.

### 2.4. Water-Sorption Measurements

Experimental data for the water-sorption curves were taken from previous studies [13,14] and are only briefly mentioned here. A drying step at 0 RH was performed 6–12 h prior to each measurement. Then, six successive stepwise changes in the RH were investigated. An RH step represents an immediate increase from a given RH to a new RH, which was then held constant until the next RH step was applied. The duration of each RH step for the measurements was terminated automatically by applying a mass-change-rate criterium < 0.0001 wt.%/min (sorption rate 1 µg/g/min).

## 3. Results

### 3.1. Anomalous Water-Sorption Kinetics in PVPVA

**Figure 2:**
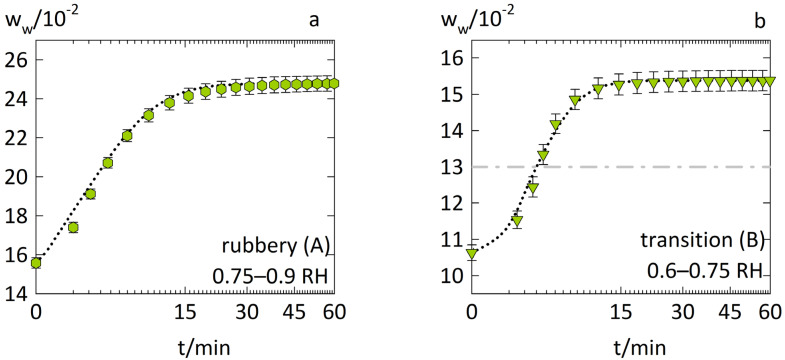
Water-sorption kinetics in PVPVA films at T = 25 °C for (**a**) step change from 0.75 to 0.9 RH (case A) as hexagons and (**b**) step change from 0.6 to 0.75 RH (case B) as downside triangles. The experimental data from previous work [13] are displayed as symbols. The modeling of the diffusion–relaxation model (Equation (1)) using the WLF equation (Equation (8)) is indicated as dotted lines. The dash-dotted line in (**b**) marks the water weight fraction at which the glass transition temperature of the PVPVA–water mixture reaches 25 °C, as derived from [31].

First, we modeled the anomalous water-sorption kinetics in PVPVA from previous work [13] using the diffusion-relaxation model in Equation (1). The square-root-of-time scaling applied in all figures of this work reveals Fickian behavior if the course of the curves for short times is linear. Figure 2a shows the water-sorption kinetics in the rubbery PVPVA during an RH step of 0.75–0.9 RH (case A). Figure 2b shows the water-sorption kinetics in PVPVA at its glass transition from 0.6–0.75 RH (case B). The water diffusion coefficient ${Đ}_{w}^{\prime \prime }$ in Table 1 was fitted to the water-sorption curve of case A (Figure 2a) using the diffusion–relaxation model (Equation (1)). The water-sorption curve of case B (Figure 2b) was then predicted using the same model and the parameters in Table 1.

The measurement and correlation of the water-sorption curve for case A were linear against the square root of time for short times. The system was above its glass transition, and the influence of polymer relaxation on diffusion was low. Thus, the water-sorption curve for case A showed mostly Fickian behavior. This supports the physical meaningfulness of the obtained water diffusion coefficient ${Đ}_{w}^{\prime \prime }$ as the water-sorption kinetics was mostly controlled by diffusion. The slight time delay observed in the measurement was likely caused by an experimental limitation as a truly instantaneous establishment of the surrounding RH was not possible.

In contrast, the water-sorption curve for case B in Figure 2b features sigmoidal characteristics with a steep upward curvature. This suggests that, at short times, water sorption was limited by polymer relaxation but accelerated by incoming water. The diffusion–relaxation model (Equation (1)) correctly predicted this accelerated water-sorption behavior, even reproducing its curvature with high quality. The diffusion–relaxation model predicted accelerated water-sorption kinetics for decreasing viscosities, as relaxation kinetics was slow for high viscosities and fast for low viscosities. The viscosity modeled by Equation (8) for case B decreased from $\sim 10^{12}\;Pa\cdot s$ to $\sim 10^{8}\;Pa\cdot s$ upon water sorption. As a result, the relaxation kinetics became faster than diffusion, which accelerated the water-sorption kinetics. The occurrence of the glass transition during case B supports this drastic change in the viscosity.

**Figure 3:**
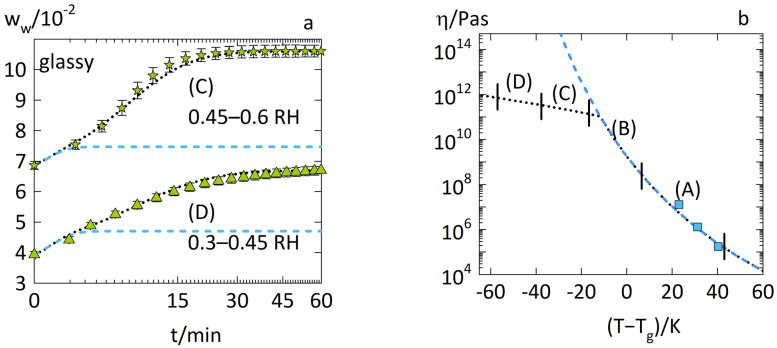
Water-sorption kinetics in PVPVA films at 25 °C during (**a**) an RH step of 0.45–0.6 RH (case C) as stars and of 0.30.45 RH (case D) as upside triangles. The experimental data from previous work [13] are displayed as symbols. The modeling of the diffusion–relaxation model (Equation (1)) using the WLF equation alone (Equation (8)) is indicated as dashed lines. The modeling of the diffusion–relaxation model (Equation (1)) using the combination of the WLF equation (Equation (8)) with the Arrhenius equation (Equation (9)) is indicated as dotted lines. The right diagram (**b**) shows the modeled viscosities of PVPVA–water mixtures via the WLF equation (Equation (8)) as a dashed line and via the combination of WLF equation (Equation (8)) and Arrhenius equation (Equation (9)) as a dotted line. The viscosities of PVPVA–water mixtures derived from the zero-shear viscosities by Wolbert et al. [16] are displayed as squares. The vertical markers denote the viscosity ranges relevant to the models of each water-sorption curve (cases A, B, C, and D, respectively).

Figure 3a shows the water-sorption kinetics in glassy PVPVA. Figure 3b shows the viscosities of the PVPVA–water mixture modeled via the WLF equation (Equation (8)) and the Arrhenius equation (Equation (9)) as a function of the distance to the glass transition temperature ($T-T_{g})$.

Figure 3a shows that the water-sorption curve from 0.45 to 0.6 RH (case C) is sigmoidal, whereas the water-sorption curve during an RH step of 0.3–0.45 RH (case D) is concave with the square root of time. Using the WLF equation alone (Equation (8)), the diffusion–relaxation model (Equation (1)) predicted a water-sorption curve that ended significantly below the experimental water weight fraction. Such behavior is modeled when the relaxation kinetics is much slower (caused by very high viscosities) than diffusion. Thus, water sorption becomes limited to a threshold, and prolonged relaxation controls further water uptake beyond this threshold until equilibrium.

However, this did not occur, and the reason for this deviation was the significant overestimation of the viscosity of glassy PVPVA–water mixtures when using the WLF equation (Equation (8)) as seen in Figure 3b for ranges C and D. The viscosities predicted via the WLF equation in this temperature range even approached infinite values, suggesting complete freezing of motions in the PVPVA–water system. Obviously, the measured water-sorption kinetics implies that there was still significant motion in the PVPVA–water system suggesting much lower viscosities than those predicted by the WLF equation. This hypothesis is supported by the excellent description of the water-sorption curve via the diffusion–relaxation model (Equation (1)) using the Arrhenius equation (Equation (9)) and fitting its parameters $E_{Ap}$ and $\Delta T_{0p}^{\alpha \beta }$ to the sorption curve (Table 1). The viscosities of the PVPVA–water mixture which resulted from this fitting (Figure 3b) were significantly lower than those predicted by the WLF equation. The modeling of all water-sorption curves experimentally obtained in our previous work [13] are displayed in Figure S2 of the Supplementary Materials.

### 3.2. Anomalous Water-Sorption Kinetics in Low-Drug-Load PVPVA–IND ASDs

**Figure 4:**
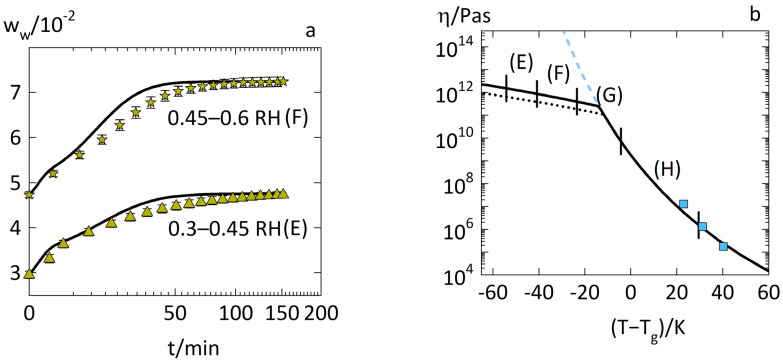
Water-sorption kinetics in PVPVA–IND ASD films at 25 °C with drug load 0.2 (**a**) step changes during RH steps of 0.3–0.45 RH (case E) as upside triangles and 0.45–0.6 RH (case F) as stars. The experimental data from previous work [14] are displayed as symbols. The modeling of the diffusion–relaxation model (Equation (1)) using Equations (9)–(11) is displayed as solid lines. (**b**) The predicted viscosities of the corresponding ASD–water mixtures, whereas the WLF equation (Equation (8)) corresponds to the dashed line and the combination of WLF equation (Equation (8)) and Arrhenius equation (Equation (9)) corresponds to the solid line. The modeled viscosities of PVPVA–water mixtures via WLF equation (Equation (8)) and Arrhenius equation (Equation (9)) are displayed as dotted lines. The experimental viscosities of PVPVA–water mixtures derived from the zero-shear viscosities by Wolbert et al. [16] are displayed as squares. The vertical markers correspond to the ranges of viscosities relevant to the models of each water-sorption curve (cases E, F, G, and H, respectively).

Figure 4a shows the predictions for the water-sorption kinetics in glassy PVPVA–IND ASDs with a drug load of 0.2 via the diffusion–relaxation model (Equation (1)) using the parameters in Table 1. Figure 4b shows the viscosities of the same ASDs with absorbed water modeled using Equation (8) until Equation (9).

Figure 4a shows that the proposed diffusion–relaxation model (Equation (1)) was able to predict the water-sorption kinetics at two different RH steps (cases E and F) in very good agreement with the experimental data using the predicted viscosities of the glassy ASD–water mixtures via Equation (9). Thus, the activation energy $E_{A}$ and distance to the switching temperature $\Delta T^{\alpha \beta }$ of the ASD–water systems were accurately predicted using the parameters for pure IND and pure PVPVA given in Table 1.

Furthermore, the water-sorption kinetics during RH steps of 0.3–0.45 RH (case E) and 0.45–0.6 RH (case F) showed a distinct kink at around 5 min which the diffusion–relaxation model (Equation (1)) accurately reproduced. This kink is the defining property of the well-known two-stage behavior. The water weight fraction at this kink is mainly determined by the elastic modulus E of the polymer as demonstrated in an earlier work [11]. Thus, the prediction of this two-stage behavior in accordance with the experimental data supports the physical significance of the elastic modulus E of PVPVA in Table 1.

**Figure 5:**
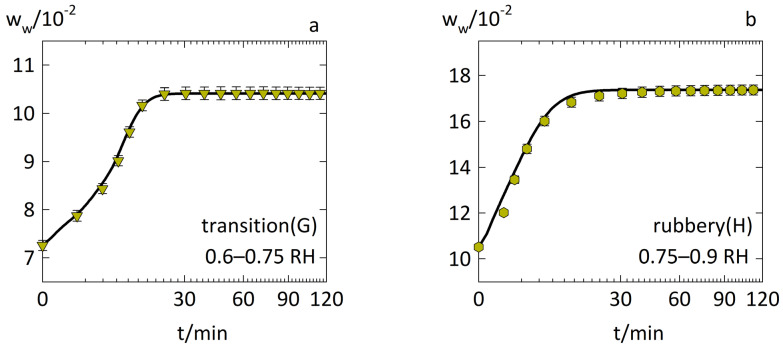
Water-sorption kinetics in PVPVA–IND ASD films at 25 °C with drug load 0.2 and RH steps of (**a**) 0.6–0.75 RH (case G) as downside triangles and (**b**) 0.75–0.9 RH (case H) as hexagons. The experimental data from previous work [14] are displayed as symbols. The modeling of the diffusion–relaxation model (Equation (1)) using the combination of WLF equation (Equation (8)) and Arrhenius equation (Equation (9)) is displayed as solid lines.

Figure 5 shows the water-sorption kinetics in the rubbery PVPVA–IND ASDs with a drug load of 0.2 as predicted via the diffusion–relaxation model (Equation (1)) using the parameters in Table 1. It accurately predicted the water-sorption kinetics, also reproducing the sharp upward curvature of the accelerated water-sorption curve for the RH step of 0.6–0.75 RH (case G) in Figure 5a and the Fickian behavior for the RH step of 0.75–0.9 RH (case H) in Figure 5b.

The prediction of case H via the diffusion–relaxation model (Equation (1)) relied on the viscosities of rubbery ASD–water mixtures modeled only via the WLF equation (Equation (8)), as seen in Figure 4b. In contrast, the prediction of case G via the diffusion–relaxation model (Equation (1)) relied on the viscosities modeled via the WLF equation (Equation (8)) and the Arrhenius equation (Equation (9)). Thus, the predicted viscosities using only the WLF equation (Equation (8)) for rubbery ASD–water mixtures (case H) and for rubbery PVPVA (Figure 2a case B) were suitable for predicting the water-sorption kinetics in these rubbery systems. Thus, the temperature–humidity–drug load correspondence principle proposed by Wolbert et al. [16] held as long as the viscosities of the polymer–water mixtures or ASD–water mixtures showed WLF behavior.

The modeling of all water-sorption kinetics in PVPVA–IND ASDs with a drug load of 0.2 from the previous work [14] is displayed in Figure S2 of the Supplementary Materials.

### 3.3. Anomalous Water-Sorption Kinetics in High-Drug-Load PVPVA–IND ASDs

**Figure 6:**
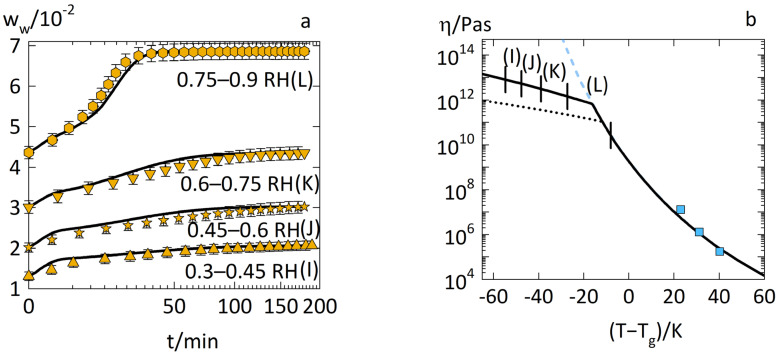
Water-sorption kinetics in PVPVA–IND ASD films at 25 °C with drug load 0.5 and (**a**) RH steps of 0.3–0.45 RH (case I) as upside triangles, 0.45–0.6 RH (case J) as stars, 0.6–0.75 RH (case K) as downside triangles, and 0.75–0.9 RH (case L) as hexagons. The experimental data from previous work [14] are displayed as symbols. The prediction via the diffusion–relaxation model (Equation (1)) using the combination of the WLF equation (Equation (8)) and Arrhenius equation (Equation (9)) is displayed as solid lines. (**b**) Viscosities of the corresponding ASD–water mixtures predicted via the WLF equation (Equation (8)) as dashed lines and via the combination of the WLF equation (Equation (8)) and Arrhenius equation (Equation (9)) as solid lines. The modeled viscosities of the PVPVA–water mixtures by the WLF equation (Equation (8)) and Arrhenius equation (Equation (9)) are displayed as dotted lines. The experimental viscosities of PVPVA–water mixtures derived from the zero-shear viscosities by Wolbert et al. [16] are displayed as squares. The vertical markers correspond to the ranges of viscosities relevant to the models of each water-sorption curve (cases I, J, K, and L, respectively).

Figure 6a shows the predictions for the water-sorption kinetics for glassy PVPVA–IND ASD with a drug load of 0.5 via the diffusion-relaxation model (Equation (1)) using the parameters in Table 1. Figure 6b shows the viscosities of the same ASDs with absorbed water modeled using Equation (8) to Equation (11).

Figure 6a shows that the water-sorption kinetics in the glassy PVPVA–IND ASDs with a drug load of 0.5, showing two-stage behavior for case I, case J, and case K. The water-sorption kinetics of case L showed an accelerated sorption behavior, as explained for PVPVA (Figure 2b case B). The prediction of these anomalous sorption behaviors via the diffusion–relaxation model (Equation (1)) using the parameters in Table 1 was excellent. Furthermore, this two-stage behavior was not observed for PVPVA but occurred for the PVPVA–IND ASD. Consequently, the API amplified the development of this anomalous sorption behavior in the ASD compared to its polymer.

Figure 6b explains this effect as the viscosities in the glassy ASD–water mixtures were higher compared to the glassy PVPVA–water mixtures when considering the same distance $\left(T-T_{g}\right)$. This result is counter-intuitive but explainable by considering the nonequilibrium free volume in the glassy ASDs compared to the glassy PVPVA. The nonequilibrium free volume is the volume that is “frozen” in a glassy state compared to its hypothetical liquid state. A high non-equilibrium free volume is associated with high localized mobility in glassy systems, which benefits diffusion and relaxation processes in glassy systems [32]. As previously demonstrated [13], the glassy PVPVA films at 25 °C had a high nonequilibrium free volume of ~8%. In contrast, glassy IND has 1–2% nonequilibrium free volume at 25 °C (based on molecular simulations by Anderson et al. [33]). Thus, introduction of IND substantially reduces the nonequilibrium free volume in the glassy ASD compared to glassy PVPVA resulting in less localized mobility in the glassy state in the ASD compared to the pure polymer. Less localized mobility results in a higher viscosity.

The predictions via the diffusion–relaxation model (Equation (1)) for all water-sorption kinetics in the PVPVA–IND ASDs with a drug load of 0.5 are shown in Figure S3 in the Supplementary Materials.

## 4. Conclusions

We modeled the anomalous water-sorption behavior of PVPVA and of PVPVA–IND ASDs at 25 °C. A diffusion–relaxation model was used to predict the anomalous water-sorption kinetics in glassy and rubbery ASDs on the basis of viscosities modeled using the WLF equation and the Arrhenius equation. In this way, viscosities of rubbery and glassy ASD–water mixtures were predicted. The water-sorption kinetics in PVPVA and ASDs featured sigmoidal characteristics with an accelerating effect on water diffusion near their glass transition. The accelerating effect was explained and predicted by the diffusion–relaxation model, suggesting a drastic decrease in the viscosity of these mixtures due to the incoming water. As a result, the water-sorption kinetics in rubbery PVPVA and rubbery ASDs showed Fickian behavior which was correctly predicted by the model.

The diffusion–relaxation model was also used to predict the water-sorption kinetics in glassy PVPVA mixtures, relying mostly on the viscosities predicted using the Arrhenius equation. The experiments revealed that anomalous water-sorption behavior was more pronounced in the glassy ASD compared to glassy PVPVA showing two-stage behavior that was not observed in the PVPVA–water system. The diffusion–relaxation model predicted this behavior in quantitative and qualitative agreement with the experimental data due to higher viscosities obtained for the glassy ASD–water mixtures compared to glassy PVPVA–water mixtures at the same distance to their glass transition temperature.

The diffusion–relaxation model is highly efficient in predicting the anomalous water sorption kinetics in ASDs for any polymer–API system, with varying drug loads and arbitrary RH steps. Thus, it provides an excellent basis for predicting the diffusion-controlled release behavior or swelling-controlled release behavior of ASDs.

## Figures and Tables

**Table 1:** Parameters used for the diffusion–relaxation model for PVPVA, water, and IND.

$\eta_{ref}{\;}^{a}$	$C_{1}{\;}^{a}$	$C_{2}{\;}^{a}$	$E_{Ap}{\;}^{b}$	$E_{Aa}{\;}^{c}$	$\Delta T_{0p}^{\alpha \beta }{\;}^{b}$	$\Delta T_{0a}^{\alpha \beta }{\;}^{d}$	$E{\;}^{e}$	${Đ}_{w}^{\prime \prime }{\;}^{b}$
/Pa·s	/-	/K	/kJ·mol^−1^	/kJ·mol^−1^	/K	/K	/GPa	m^2^·s^−1^
1.83 × 10^5^	10.04	147.4	41.29	79	−12.31	−20	4	3.93 × 10^−13^

*^a^* taken from Wolbert et al. [16]; *^b^* fitted to the water-sorption kinetics in PVPVA in this study; *^c^* taken from Correia et al. [27] measured by thermally stimulated depolarization currents of IND; *^d^* taken from Andronis and Zografi [28]; *^e^* fitted to low-temperature storage modulus of PVP by Cassu and Felisberti [29].

## Data Availability

Data are contained within the article or Supplementary Materials.

## Supplementary Materials

The following supporting information can be downloaded at https://www.mdpi.com/article/10.3390/pharmaceutics14091897/s1: Equation (S1); Figure S1. Determination of the modulus; Figure S2. Models and predictions of all water-sorption kinetics in PVPVA and PVPVA–IND ASDs; Figure S3. Predictions for water-sorption kinetics in PVP–IND ASDs. References [13,14,29] are cited in the Supplementary Materials.
