# Supplementary material for: Anomalous Water-Sorption Kinetics in ASDs
Source: Pharmaceutics. 2022 Sep 7;14(9):1897. doi: 10.3390/pharmaceutics14091897 (PMC9505135; doi:10.3390/pharmaceutics14091897)
Supplement: Supplementary file 1 [file pharmaceutics-14-01897-s001.zip › pharmaceutics-1863827-supplementary.pdf]

pharmaceutics

MDPI

Supplementary Material

Anomalous Water-sorption Kinetics in ASDs

Dominik Borrmann, Andreas Danzer and Gabriele Sadowski *

Laboratory of Thermodynamics, Department of Chemical and Biochemical Engineering, TU Dortmund University, Emil-Figge-Str. 70, 44227 Dortmund, Germany

* Correspondence: gabriele.sadowski@tu-dortmund.de; Tel.: +49-231-755-2635

Elastic modulus

We used dynamic mechanical analysis data taken from Cassu and Felisberti [1] of PVP at 1Hz to obtain the elastic modulus E. We assume that the glassy modulus of PVP and PVPVA is the same as PVPVA is a copolymer of PVP. The storage modulus E' was described by Equation (S1), which is the analytical solution of Equation (2) from the main manuscript for a sinusoidal input of the strain ϵ that is applied in a dynamic mechanical analysis.

$$E' = E\frac{(\eta/E)^2 t^{-2}}{1-(\eta/E)^2 t^{-2}} \quad \text{(S1)}$$

Here, E is the elastic modulus, η is the viscosity, and t is the time determined from the inverse of the frequency of the sinusoidal input of the strain ϵ. The description and measurements are displayed in **Error! Reference source not found.**.

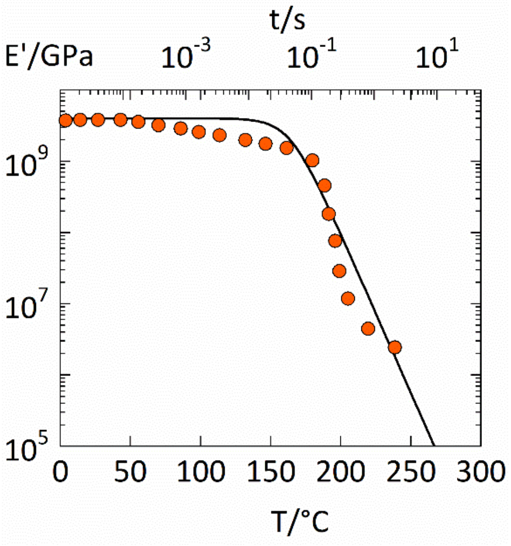

Figure S1. Dynamical mechanical analysis result from literature and modeling. Storage moduli at different temperatures were taken from Cassu and Felisberti [1] (circles) at a frequency of 1Hz. The solid lines correspond to the upper time-Axis and show the calculation from Equation (S1).

The storage moduli converge at temperatures below 50°C to a nearly constant value of ~4 GPa. The time-temperature correspondence principle states that the time and temperature dependencies of the storage modulus E' are the same. This way, E' at low temperature corresponds to E' at low times. Thus, the elastic modulus corresponds to the

low-temperature modulus of these polymers, and the elastic modulus $E = 4GPa$ was obtained.

Modeling of all water sorption curves

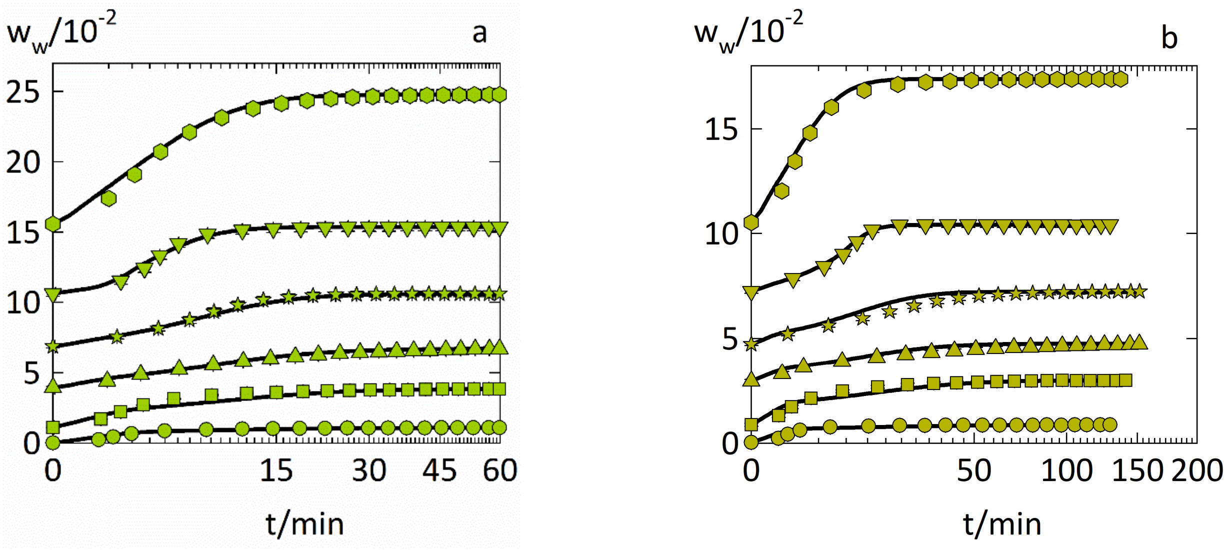

Figure S2. Water-sorption kinetics in PVPVA(a) and PVPVA-IND ASD films(b) with a drug load of 0.2 during an RH step 0-0.1 RH(circles), 0.1-0.3 RH (squares), 0.3-0.45 RH (downside triangles), 0.45-0.6 RH (stars), 0.6-0.75 RH (upside triangles) and 0.75-0.9 RH(hexagons) on the right taken from [2],[3]. The solid lines indicate the prediction by the diffusion-relaxation model (Equation (1)) using the combination of WLF equation (Equation (8)) and Arrhenius equation (Equation (9)) of the main manuscript .

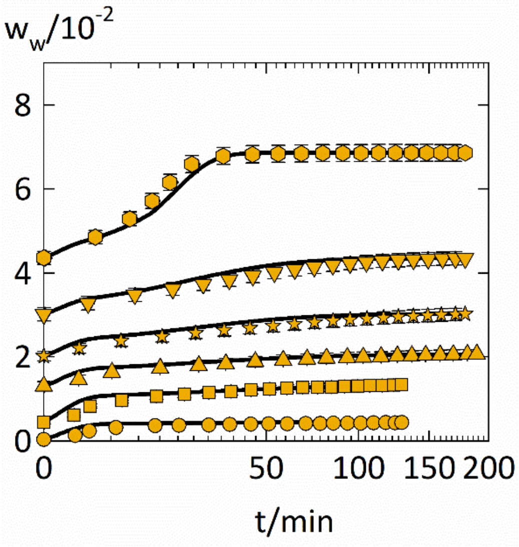

Figure S3. Water-sorption kinetics in PVPVA-IND ASD films with a drug load of 0.5 during an RH step 0-0.1 RH(circles), 0.1-0.3 RH (squares), 0.3-0.45 RH (downside triangles), 0.45-0.6 RH (stars), 0.6-0.75 RH (upside triangles) and 0.75-0.9 RH(hexagons) with data taken from[3]. The solid lines indicate the prediction via the diffusion-relaxation model (Equation (1)) using the combination of (Equation (8)) and (Equation (9)).

References

1. Cassu, S.N.; Felisberti, M.I. Poly(Vinyl Alcohol) and Poly(Vinylpyrrolidone) Blends: 2. Study of Relaxations by Dynamic Mechanical Analysis. *Polymer (Guildf).* **1999**, *40*, 4845–4851, doi:10.1016/S0032-3861(98)00703-4.
2. Borrmann, D.; Danzer, A.; Sadowski, G. Water Sorption in Glassy Polyvinylpyrrolidone-Based Polymers. *Membranes (Basel).* **2022**, *12*, 434, doi:10.3390/membranes12040434.
3. Borrmann, D.; Danzer, A.; Sadowski, G. Predicting the Water Sorption in ASDs. *Pharmaceutics* **2022**, *14*, 1181, doi:10.3390/pharmaceutics14061181.
